# Liquid chromatography tandem mass spectrometry method for the quantification of vandetanib in human plasma and rat liver microsomes matrices: metabolic stability investigation

**DOI:** 10.1186/s13065-017-0274-4

**Published:** 2017-05-30

**Authors:** Sawsan M. Amer, Adnan A. Kadi, Hany W. Darwish, Mohamed W. Attwa

**Affiliations:** 1 Analytical Chemistry Department, Faculty of Pharmacy, Cairo University, Kasr El-Aini St., Cairo, 11562 Egypt; 2 Department of Pharmaceutical Chemistry, College of Pharmacy, King Saud University, P.O. Box 2457, Riyadh, 11451 Kingdom of Saudi Arabia

**Keywords:** Vandetanib, Quantification, Tandem mass spectrometry, Human plasma, Rat liver microsomes, Metabolic stability

## Abstract

Vandetanib (VNT) is a new oral tyrosine kinase inhibitor that acts mainly by inhibiting vascular endothelial growth factor receptor (VEGFR). Fast, specific, sensitive and validated LC–MS/MS was established for the determination of VNT in two various matrices including rat liver microsomes (RLMs) and human plasma. This method was applied in metabolic stability investigation of VNT. Resolution of two analytes was performed using C18 column and isocratic mobile phase composed of binary system of 10 mM ammonium formate (pH 4.1) and acetonitrile in a ratio of 1:1. The flow rate was set at 0.25 mL/min and total run time was 4 min with injection volume of 5 µL. Ions were generated by ESI source and analyzed by multiple reaction monitoring mode (basis for quantification) in the Agilent 6410 QqQ analyzer. The linearity of the established method ranged from 5 to 500 ng/mL (r^2^ ≥ 0.9996) in human plasma and RLMs. *LOQ* and *LOD* were 2.48 and 7.52 ng/mL, and 2.14 and 6.49 in human plasma and RLMs matrices. The intra-day and inter-day precision and accuracy were 0.66–2.66% and 95.05–99.17% in human plasma matrix while in RLMs matrix, ranged from 0.97 to 3.08% and 95.8 to 100.09%, respectively. In vitro half-life was 39.85 min and intrinsic clearance was 3.92 ± 0.28 mL/min/kg.

## Background

Cancer is one of the leading reasons of death that results in More than one-fourth of the world’s deaths [1]. The management of disseminated cancer have lately been done by molecular targeting strategies, based on the examinations of the oncogenes and tumor suppressors contributed in the development of human cancers [2]. Tyrosine kinase inhibitors (TKIs) are an imperative novel class of targeted therapy which interfere with specific cell signalling pathways and hence permit target specific therapy for selected malignancies [3].

**Fig. 1:**
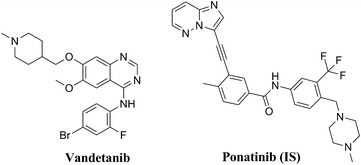
Chemical structure of Vandetanib and Ponatinib (IS)

VNT (Fig. 1) is a vascular endothelial growth factor receptor 2 (VEGFR) inhibitor [4]. VEGFR has gained importance as pharmacologic targets as a Tyrosine kinase receptors [5]. In 2011, VNT (Caprelsa^®^ tablets; AstraZeneca Pharmaceuticals LP) was approved by the USFDA for management of various types of medullary thyroid cancer. It was the first drug approved for this case. Its toxicity profile includes prolongation of the QT interval and sudden death [6].

The goal of our work is to study the metabolic stability and clearance of VNT, and accordingly a new LC–MS/MS method was established. Examining the literature showed that there were three reported methods to quantify VNT in human plasma by LC–ESI–MS/MS [7], HPLC–UV [8] and spectrofluorometry. In the LC–ESI–MS/MS method, the linearity was 1.0–3000 ng/mL but the recovery % of VNT was around 80%. In the HPLC–UV method, the linearity range was from 80 to 4000 ng/mL. In the third one, the linearity was ranged from 20 to 600 ng/mL [9]. No publication was reported about quantification of VNT in RLMs matrix or the study of VNT metabolic stability. Therefore, these results motivated us for development of an efficient and validated method for estimation of VNT level with high accuracy and precision. Accordingly, an LC–MS/MS technique was adopted for measurement of VNT concentration in human plasma and RLMs matrices. The current procedure gave higher recovery than the reported LC–MS/MS (around 99% compared with 80% for the reported one), additionally, our method sensitivity is higher than the other two reported methods as our linearity range was 5–500 ng/mL.

The proposed method is applied for assessing the metabolic stability of in RLMs depending on the rate of disappearance of the drug during its incubation with RLMs. In vitro half-life (t_1/2_) and intrinsic clearance (CL_int_) were utilized for expressing of metabolic stability and hence hepatic clearance (CL_H_), bioavailability and in vivo t_1/2_ can be calculated. If a test compound is rapidly metabolized, its in vivo bioavailability will probably be low [10].

## Experimental

### Chemicals and reagents

All solvents were of HPLC grade and reference powders were of analytical grade. Vandetanib and ponatinib were procured from LC Laboratories (Woburn, MA, USA). Formic acid, ammonium formate, and ACN were procured from Sigma-Aldrich (West Chester, PA, USA). HPLC water grade was generated by in house Milli-Q plus purification system (Millipore, Waters, USA). Preparation of RLMs was done in house using Sprague Dawely rats [11]. Human plasma was kindly gifted by King Khalid University Hospital (Riyadh, KSA). After informed consent was gotten, fasting blood samples were taken and plasma was separated and stored at −70 °C.

### Chromatographic conditions

An Agilent 6410 LC–MS/MS (Agilent Technologies, Palo Alto, CA, USA) was utilized for separation of VNT (analyte) and IS. HPLC was Agilent 1200 LC system. Mass analyzer was Agilent 6410 triple quadrupole (QqQ MS) with an ESI interface. Separation of VNT and IS was done using C_18_ column (Agilent eclipse plus) with 50 mm length, 2.1 mm internal diameter and 1.8 μm particle size. Temperature of the column was adjusted at 22 ± 1 °C. All chromatographic parameters were adjusted to attain the best resolution in a short time. A pH value was adjusted at 4.1 as above this value a remarked increase in retention time and a tailing were observed. The ratio of ACN to aqueous phase was adjusted to 1: 1, as increasing ACN led to bad separation and overlapped peaks. On contrary, decreasing ACN percent lead to unnecessary delayed retention time. Different columns such as Hilic column were tested and the cited analytes were not retained. The best results were accomplished using C_18_ column and isocratic mobile phase composed of binary system of 10 mM ammonium formate (*p*H: 4.1) and acetonitrile (ACN) in a ratio of 1:1. The flow rate was set at 0.25 mL/min and total run time was 4 min with injection volume of 5 µL. Mass parameters were optimized for VNT and IS. Ions were generated in positive ESI source, analyzed by 6410 QqQ mass spectrometer and detected by mass detector. Nitrogen gas was utilized for drying (flow rate = 11 L/min) and nitrogen of high purity was used as a collision gas in the collision cell (pressure = 50 psi). Source temperature and capillary voltage were kept at 350 °C and 4000 V, respectively. Mass Hunter software was utilized for data acquisition. Quantitation was accomplished using multiple reactions monitoring (MRM) for the transition 475→112 in case of VNT, and for transitions 533→433 and 533→260 in case of IS. Fragmentor voltage was adjusted at 145 V with collision energy of 15 eV for VNT and 140 and 145 V with collision energy of 16, 15 eV for IS.

### Preparation of standard solutions

One mg/mL stock solution of VNT was prepared in DMSO then diluted 10-folds with the mobile phase to give working solution 1 (WK1, 100 µg/mL). One mL of WK1 was diluted 10-folds with mobile phase to give working solution 2 (WK2, 10 µg/mL). Stock solution (100 µg/mL) of IS was prepared in DMSO then 200 µL of this solution was diluted to 10 mL with the mobile phase to give working solution 3 (WK3, 2 µg/mL).

### Preparation of RLMs matrix

Four rats (Sprague–dawley) were supplied by the experimental animal care center at college of pharmacy, King Saud University (Riyadh, KSA). The animal experimental protocol was approved by the University’s Ethics Review Committee. First, cervical dislocation of the rats was done then an incision was made in the peritoneal cavity. The rats’ livers were then removed and transferred to clean beaker and weighed. Ice-cold KCl/sucrose buffer (containing 0.04 M KH_2_PO_4_/NaH_2_PO_4_, 0.25 M sucrose, 0.15 M KCl, pH 7.4) was added to the rat liver in a ratio of 1/4 W/V. Liver was cut to small pieces then homogenized using OMNI homogenizer. Two steps of centrifugation were done for the liver homogenate. The first step of centrifugation was done at 9000*g* for 25 min at 4 °C to get S9 which is the supernatant. The second step for centrifugation was done for the supernatant at a 100,000*g* for 65 min. Then, the supernatant was removed while pellets (RLMs) were suspended in KCl/sucrose buffer. Storing of RLMs suspension was done in a deep freezer at −76 °C. Lowry method [12] was adopted for protein content determination of RLMs.

### Sample preparation and generation of the calibration curve

Human plasma or RLMs matrix was spiked with proper volumes of VNT WK2 (10 µg/mL) to produce two sets of twelve concentrations: 5, 10, 20, 30, 40, 50, 80, 100, 150, 300, 400 and 500 ng/mL. Three concentrations (20, 150 and 400 ng/mL) were selected as low quality control (LQC), medium quality control (MQC) and high quality control (HQC), respectively. One mL of 0.1 M NaOH/glycine buffer (pH 9.5) was added to all samples followed by vortexing for 30 s then 2 mL of ACN was added for protein precipitation. Centrifugation at 14,000 rpm (12 min at 4 °C) was done to get rid of precipitated proteins. Filtration of the supernatants was done through 0.22 µm syringe filter. IS (50 µL) was added to 1 mL of the filtered standards and 5 µL were injected into LC–MS/MS. The same procedure was applied to prepare blank using mobile phase instead to confirm the absence of any interference at the retention time of VNT and IS. Two Calibration curve (5, 10, 20, 30, 40, 50, 80, 100, 150, 300, 400 and 500 ng/mL) were created for spiked human plasma and RLMs samples by drawing the peak area ratio of VNT to IS (*y* axis) versus VNT concentrations (*x* axis). Different parameters including slope, intercept, and r^2^ values were computed for expressing linear regression.

### Method validation

Validation of the analytical method was done following the general recommendations of International Conference on Harmonisation (ICH) [13] and the guidelines for analytical procedures and methods validation by the FDA [14].

### Specificity

To study the specificity of the suggested analytical method, six separate blank RLMs and human plasma matrices samples were treated with the proposed extraction technique. Those samples were then analyzed for any interfering peaks at retention time of VNT or IS and comparing the chromatogram with VNT and IS spiked human plasma and RLMs matrices samples. MRM mode in the mass analyzer was used to minimize carryover effects.

### Linearity and sensitivity

Six various calibration curves in each matrix were established to calculate linearity and sensitivity of the suggested method. Calibration samples were freshly prepared daily at twelve concentration levels ranging from 5 to 500 ng/mL. Analysis of results were done using statistical least square method. Limit of detection (LOD) and limit of quantitation (LOQ) were computed following the ICH guidelines [13].

### Precision and accuracy

Intra-day precision and accuracy were calculated by the analysis of different matrices samples spiked with VNT and QC levels in 1 day. Additionally, inter-day measurements were done on three consecutive days. Percentages accuracy (100—% RE) and percentages relative standard deviation (% RSD) were used to express accuracy and precision of the established methods, respectively.

### Assay recovery

Extraction recovery of VNT was evaluated by comparing the mean peak area of VNT in the QC samples with the mean peak area of VNT extracted from blank plasma or blank RLM that spiked with correspondent VNT reference solutions (n = 5).

### Stability

For determination of VNT stability in different matrices, analysis of six replicates of QC samples were performed under various storage conditions. Accuracy and precision values were computed using data generated form fresh prepared human plasma and RLMs calibration curves were u. VNT QC samples were kept at room temperature for 8 h to estimate VNT bench-top stability. Three freeze–thaw cycles were done to determine VNT stability of spiked QC samples after freezing them at −76 °C and thawing them at room temperature. Additionally, determination of VNT stability was achieved by analyzing the spiked QC samples after keeping them at 4 °C for 1 day and after their storage at −20 °C for 30 days.

### Metabolic stability of VNT

The decrease in VNT concentration after incubation with RLMs matrix was utilized to study the metabolic stability of VNT. Incubations of 1 µM VNT with 1 mg/mL microsomal proteins were done in triplicates. Pre incubation for all samples was done for 10 min to attain 37 °C. The metabolic reaction was initiated by adding 1 mM NADPH in phosphate buffer (pH 7.4) containing 3.3 mM MgCl_2_ and terminated by adding 2 mL of ACN at time intervals of 0, 2.5, 5, 10, 15, 20, 40, 50, 70, 90 and 120 min. The extraction of VNT was done following the same sample preparation procedure as above. Concentrations of VNT in RLMs matrix were computed from the regression equation of freshly prepared calibration curve of VNT.

## Results and discussion

### Chromatographic separation and mass spectrometry

Chromatographic and mass spectrometric parameters were adjusted to attain the most stable mass response and increase the resolution and sensitivity. *p*H of solvent A (aqueous portion) enhanced VNT ionization and helped in the adjustment of peak shape. Different percentages of the mobile phase were examined. Binary isocratic mobile phase system was used for separation of VNT and IS. System composition was ACN and 10 mM ammonium formate buffer (pH ~4.1 adjusted by addition of formic acid) in a ratio of 1:1. VNT and IS were eluted at retention times of 1.3 and 2.5 min, respectively.

**Fig. 2:**
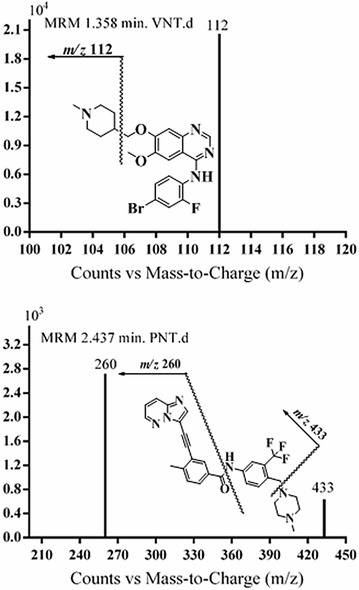
MRM mass spectra of (**a**) VNT and (**b**) IS

MRM mode was utilized in our work to remove any probable interference from matrices components and elevate the method sensitivity. MS scan spectra of VNT and IS consisted mainly of a single molecular ion (MI) at m/z 475 (VNT) and at m/z 533 (IS). Fragmentation of VNT MI at m/z 475 gave one product ion at m/z 112. Similarly, fragmentation of IS MI at m/z 533 gave product ions at m/z 433 and 260. Those ions were chosen for the MRM mode for VNT and IS in the quantification method (Fig. 2).

**Fig. 3:**
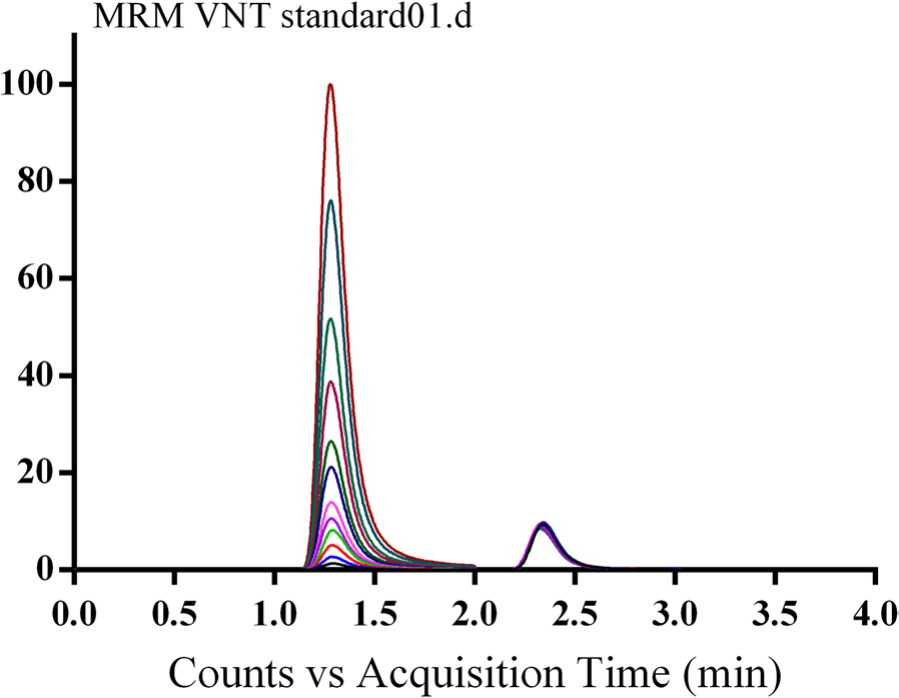
Overlayed TIC chromatograms of MRM of VNT (5–500 ng/mL) and IS (50 ng/mL)

The separation of VNT and IS was attained in 4 min. VNT and IS peaks were well separated, with no carryover in any blank matrix (RLMs or plasma) sample or VNT-free standard (blank + internal standard). Figure 3 showed overlayed MRM chromatograms of calibration standard solutions.

## Method validation

### Specificity

**Fig. 4:**
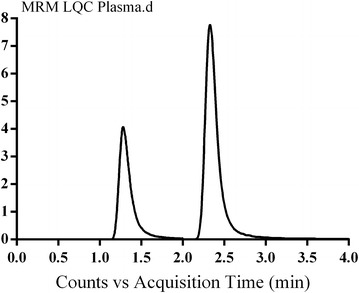
Overlayed MRM chromatogram of VNT LQC in plasma and blank plasma showing no interference from plasma matrix

**Fig. 5:**
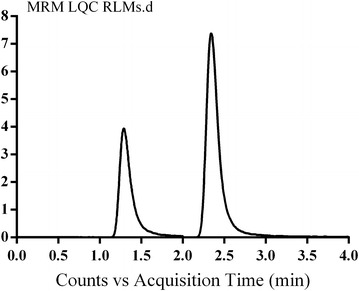
Overlayed MRM chromatogram of VNT LQC in RLMs and blank RLMs showing no interference from RLMs matrix

The established LC–MS/MS method was specific as there were no interference from constituents of both matrices at the elution time of VNT and/or IS (Figs. 4, 5). No carry over effect of analytes was noticed in the MS detector. VNT and IS chromatographic peaks were well separated under the adjusted conditions with retention times of 1.3 and 2.5 min, respectively.

### Linearity and sensitivity

The established LC–MS/MS was rugged and sensitive for VNT analysis in human plasma and RLMs matrices. The least-square method was utilized for analyzing the linear regression results. Linearity range was 5–500 ng/mL, and the correlation coefficients (r^2^) ≥ 0.9996 in human plasma and RLMs matrices. The regression equations of calibration curves of VNT in human plasma and RLMs were y = 2.726x + 2.227 and y = 2.747x + 2.133, respectively. LOD and LOQ were equal to 2.48 and 7.52 ng/mL, and 2.14 and 6.49 ng/mL in human plasma and RLMS matrices, respectively.

The RSD values of each concentration point (six repeats) did not exceed 6.4 and 3.99% in human plasma and RLMs matrices, respectively. Calibration and QC samples of VNT in both matrices (twelve points) were back-calculated to ensure the best performance of the developed method. The precision and accuracy were 1.07–4.82% and 98.9 ± 2.54%, in human plasma matrix, respectively (Table 1), while were ranged from 0.28 to 4.32% and 99.4 ± 2.56% in RLMs matrix, respectively. The mean recoveries percent of VNT were 98.9 ± 2.5% and 99.12 ± 4.48% in human plasma and RLMs matrices, respectively.

**Table 1:** Data of back-calculated VNT concentration of the calibration standards from human plasma and RLMs matrices

Nominal concentration (ng/mL)	Human plasma			RLMs		
	Mean^a^ ± SD	Precision (% RSD)	% Accuracy	Mean^a^ ± SD	Precision (% RSD)	% Accuracy
5	4.77 ± 0.19	3.89	95.40	4.79 ± 0.17	3.45	95.80
10	9.49 ± 0.25	2.66	94.90	9.54 ± 0.19	2.02	95.40
20	19.36 ± 0.43	2.22	96.80	19.47 ± 0.31	1.61	97.35
30	29.27 ± 0.48	1.63	97.57	29.43 ± 0.59	2.01	98.10
40	41.00 ± 1.26	3.06	102.50	41.22 ± 1.18	2.85	103.05
50	51.05 ± 2.46	4.82	102.10	51.31 ± 2.22	4.32	102.62
80	79.32 ± 1.61	2.03	99.15	79.74 ± 1.21	1.52	99.68
100	100.92 ± 1.32	1.31	100.92	101.46 ± 0.91	0.90	101.46
150	150.78 ± 1.29	0.86	100.52	151.59 ± 0.50	0.33	101.06
300	290.64 ± 3.31	1.14	96.88	292.22 ± 3.99	1.37	97.41
400	400.38 ± 6.40	1.60	100.10	402.51 ± 3.47	0.86	100.63
500	499.05 ± 5.34	1.07	99.81	501.72 ± 1.41	0.28	100.34

^a^Average of six determinations

### Precision and accuracy

Reproducibility was confirmed by intra- and inter-day precision and accuracy at QC concentrations. Accuracy and precision values lied into the allowed range following ICH guidelines [15, 16] as seen in Table 2.

**Table 2:** Intra-day and inter-day precision and accuracy of the proposed methods

	LQC (20 ng/mL)		MQC (150 ng/mL)		HQC (400 ng/mL)	
	Intra-day assay^a^	Inter-day assay^b^	Intra-day assay	Inter-day assay	Intra-day assay	Inter-day assay
Human plasma matrix						
Mean	19.01	19.22	148.71	148.59	397.28	396.68
Standard deviation (SD)	0.50	0.45	1.54	1.55	2.61	3.02
Precision (% RSD)	2.66	2.36	1.04	1.04	0.66	0.76
% Accuracy	95.05	96.1	99.14	99.06	99.32	99.17
RLMs matrix						
Mean	19.16	19.27	149.80	150.14	398.55	399.02
Standard deviation (SD)	0.59	0.47	1.84	1.45	5.36	5.42
Precision (% RSD)	3.08	2.44	1.23	0.97	1.34	1.36
% Accuracy	95.80	96.35	99.87	100.09	99.64	99.76

^a^Average of twelve determinations of day 1

^b^Average of six determinations in three consecutive days

### Extraction recovery and matrix effects

QC samples extraction recoveries were shown in Table 3. The recoveries of VNT were 99.14 ± 2.04% (human plasma) and 99.68 ± 2.03% (RLMs). To confirm the lack of matrix effect on the VNT analysis, 6 various batches of both matrices were extracted and spiked with 20 ng/mL of VNT (LQC) and IS as set 1. Similarly, preparation of set 2 was performed, which consisted of 6 replicates of same concentrations of VNT and IS but solubilized in mobile phase. For estimation of matrix effect, mean peak area ratio of set 1/set 2 × 100 was calculated. The studied plasma and RLMs matrices containing VNT showed 95.63 ± 2.55% and 96.9 ± 1.12%, respectively. Accordingly, these results exhibited that plasma and RLMs matrices have little impact on the ionization of VNT and PNT (IS).

**Table 3:** Recovery of quality control samples for determining the concentration of VNT in human plasma and RLMs matrices

Nominal concentration (ng/mL)	Human plasma			RLMs		
	20 ng/mL	150 ng/mL	400 ng/mL	20 ng/mL	150 ng/mL	400 ng/mL
Mean^a^	19.36	150.78	400.38	19.47	151.59	402.51
Recovery (%)	96.80	100.52	100.10	97.35	101.06	100.63
Standard deviation (SD)	0.43	1.29	6.4	0.31	0.5	3.47
Precision (% RSD)	2.22	0.86	1.6	1.61	0.33	0.86

^a^Average of six determinations

### Stability

Stability experiments were done using QC samples. Stability of VNT was tested under various conditions. SD of the results from the average value of samples of human plasma and RLMs matrices was less than 4.82 and 4.32%, respectively. No observed loss of VNT happened during sample storage and handling under the examined conditions. Stability results (Tables 4, 5) approved that matrices samples (human plasma or RLMs) containing VNT can be retained under laboratory conditions with no noticeable change of its concentration.

**Table 4:** VNT stability data in plasma matrix under different conditions

Nominal concentration (ng/mL)	Mean (ng/mL)	Recovery %	Precision (% RSD)
Room temp. for 8 h			
20	18.77 ± 0.58	93.84	3.08
150	148.23 ± 1.84	98.82	1.24
400	396.81 ± 5.55	99.20	1.40
Three freeze–thaw cycles			
20	18.98 ± 0.42	94.89	2.22
150	150.03 ± 1.28	100.02	0.86
400	397.17 ± 6.35	99.29	1.60
Stored at 4 °C for 24 h			
20	19.00 ± 0.32	95.02	1.70
150	149.06 ± 1.55	99.37	1.04
400	397.22 ± 5.92	99.31	1.49
Stored at −20 °C for 30 days			
20	19.00 ± 0.32	94.98	1.67
150	147.82 ± 0.96	98.55	0.65
400	395.48 ± 4.69	98.87	1.19

**Table 5:** VNT stability data in RLMs matrix under different conditions

Nominal concentration (ng/mL)	Mean (ng/mL)	Recovery %	Precision (% RSD)
Room temp. for 8 h			
20	19.35 ± 0.28	96.75	1.45
150	148.71 ± 1.62	99.14	1.09
400	396.96 ± 3.15	99.24	0.79
Three freeze–thaw cycles			
20	19.46 ± 0.36	97.31	1.86
150	150.47 ± 1.16	100.32	0.77
400	399.58 ± 6.02	99.89	1.51
Stored at 4 °C for 24 h			
20	19.43 ± 0.33	97.14	1.70
150	150.26 ± 0.98	100.17	0.65
400	398.08 ± 4.73	99.52	1.19
Stored at −20 °C for 30 days			
20	19.16 ± 0.59	95.78	3.08
150	149.80 ± 1.84	99.87	1.23
400	398.55 ± 5.36	99.64	1.34

### Metabolic stability

**Fig. 6:**
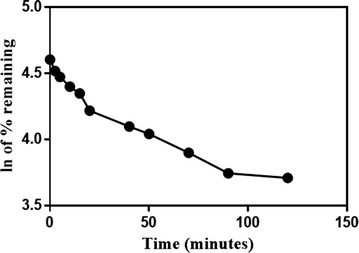
The metabolic stability profile of VNT

The metabolic reaction of VNT and RLMs was quenched at specific time points. The ln of the % remaining of VNT concentration (comparing to zero-time concentration) was plotted against time of incubation as shown in Fig. 6. In vitro t_1/2_ was computed from the regression equation of the linear part of the curve [15]. The slope was 0.017 so in vitro t_1/2_ was 39.85 min according to the following formula.

$${\text{In vitro}}\;t _{1/2} = {\raise0.7ex\hbox{${ln2}$} \!\mathord{\left/ {\vphantom {{ln2} {Slope}}}\right.\kern-0pt} \!\lower0.7ex\hbox{${Slope}$}}$$

$${\text{In vitro}} \;t _{1/2} = 39.85\; min.$$

Consequently, CL_int_ (3.92 ± 0.28) was computed according to in vitro t_1/2_ method [10] as anticipated in the next formula:

$$\begin{aligned} CL_{int, app} = \frac{0.693}{{{\text{in vitro}}\; t _{1/2} }} & . \frac{\text{mL incubation}}{\text{mg microsomes}} \\ & .\frac{{45\,{\text{mg microsome}}}}{\text{g liver}} .\frac{{20\, {\text{g liver}}}}{\text{kg per body weight}} \\ \end{aligned}$$

$$CL_{int, app} = \frac{0.693}{39.85} . \frac{1}{1} .\frac{45}{12.5} .\frac{20 }{0.32}$$

$$CL_{int, app} = 3.91\; {\text{mL}}/{ \hbox{min} }/{\text{kg}}$$

The low intrinsic capacity of RLMs to metabolize VNT (CL_int_ = 3.91 mL/min/kg) with long in vitro t_1/2_ (approximately 40 min) suggested that VNT is slowly cleared from the blood by the liver and thus considered as low extraction ratio drug. The low intrinsic capacity of liver to metabolize VNT is a specific character for the cited drug not a general feature to similar TKIs as when we investigated the CL_int_ and in vitro t_1/2_ of ponatinib in our previous article [16], we found that CL_int_ was 15 mL/min/kg with short in vitro t_1/2_ of approximately 6 min.

## Conclusions

LC–MS/MS method was established for estimation of VNT concentration in different matrices including human plasma and RLMs. This method is simple, sensitive, and rapid with linearity range of 5–500 ng/mL and LOD of 2.48 and 2.14 ng/mL in human plasma and RLMs, respectively. The established procedure characterized by consumption of small volume of solvents (flow rate = 0.25 mL/min.) and fast run time (4 min.). The recovery of VNT from human plasma and RLMs was 99.14 ± 2.04% and 99.68 ± 2.03%. The established procedure was useful for the assessment of VNT metabolic stability. In vitro t_1/2_ (39.85 min) and intrinsic clearance (3.91 mL/min/kg) were utilized to express VNT metabolic stability. The low intrinsic capacity of RLMs to metabolize VNT (CL_int_ = 3.91) with longer in vitro t_1/2_ of approximately 40 min suggests that VNT is slowly cleared from the blood by the liver and thus considered as low extraction ratio drug.

## References

[1] Jemal A, Siegel R, Ward E, Hao Y, Xu J, Murray T (2008). Cancer statistics, 2008. CA Cancer J Clin.

[2] Barinaga M (1997). From bench top to bedside. Science.

[3] Natoli C, Perrucci B, Perrotti F, Falchi L, Iacobelli S (2010). Tyrosine kinase inhibitors. Curr Cancer Drug Targets.

[4] Martin P, Oliver S, Kennedy SJ, Partridge E, Hutchison M, Clarke D (2012). Pharmacokinetics of vandetanib: three phase I studies in healthy subjects. Clin Ther.

[5] Li F, Jiang S, Zu Y, Lee DY, Li Z (2014). A tyrosine kinase inhibitor-based high-affinity PET radiopharmaceutical targets vascular endothelial growth factor receptor. J Nucl Med.

[6] Thornton K, Kim G, Maher VE, Chattopadhyay S, Tang S, Moon YJ (2012). Vandetanib for the treatment of symptomatic or progressive medullary thyroid cancer in patients with unresectable locally advanced or metastatic disease: US Food and Drug Administration drug approval summary. Clin Cancer Res.

[7] Bai F, Johnson J, Wang F, Yang L, Broniscer A, Stewart CF (2011). Determination of vandetanib in human plasma and cerebrospinal fluid by liquid chromatography electrospray ionization tandem mass spectrometry (LC–ESI–MS/MS). J Chromatogr B Anal Technol Biomed Life Sci.

[8] Lin H, Cui D, Cao Z, Bu Q, Xu Y, Zhao Y (2014). Validation of a high-performance liquid chromatographic ultraviolet detection method for the quantification of vandetanib in rat plasma and its application to pharmacokinetic studies. J Cancer Res Ther.

[9] Darwish HW, Bakheit AH (2016). A new spectrofluorimetric assay method for vandetanib in tablets, plasma and urine. Trop J Pharm Res.

[10] Baranczewski P, Stanczak A, Sundberg K, Svensson R, Wallin A, Jansson J (2006). Introduction to in vitro estimation of metabolic stability and drug interactions of new chemical entities in drug discovery and development. Pharmacol rep PR.

[11] von Jagow R, Kampffmeyer H, Kiese M (1965). The preparation of microsomes. Naunyn-Schmiedebergs Archiv fur experimentelle Pathologie und Pharmakologie.

[12] Lowry OH, Rosebrough NJ, Farr AL, Randall RJ (1951). Protein measurement with the Folin phenol reagent. J Biol Chem.

[13] Guideline IHT (2005) Validation of analytical procedures: text and methodology. Q2 (R1). 2005;1

[14] Fda AP (2000). Methods validation: chemistry, manufacturing and controls documentation availability. Federal Regist (Notices).

[15] Caldwell G, Yan Z (2014). Optimization in drug discovery: in vitro methods.

[16] Kadi AA, Darwish HW, Attwa MW, Amer SM (2016). Validated LC–MS/MS method for the quantification of ponatinib in plasma: application to metabolic stability. PLoS ONE.

